# kmacs: the *k*-mismatch average common substring approach to alignment-free sequence comparison

**DOI:** 10.1093/bioinformatics/btu331

**Published:** 2014-05-13

**Authors:** Chris-Andre Leimeister, Burkhard Morgenstern

**Affiliations:** ^1^Department of Bioinformatics, Institute of Microbiology and Genetics, University of Göttingen, Goldschmidtstr. 1, 37073 Göttingen, Germany and ^2^Laboratoire Statistique et Génome, Université d’Évry Val d’Essonne, UMR CNRS 8071, USC INRA, 23 Boulevard de France, 91037 Évry, France

## Abstract

**Motivation:** Alignment-based methods for sequence analysis have various limitations if large datasets are to be analysed. Therefore, alignment-free approaches have become popular in recent years. One of the best known alignment-free methods is the *average common substring approach* that defines a distance measure on sequences based on the average length of longest common words between them. Herein, we generalize this approach by considering longest common substrings with *k* mismatches. We present a greedy heuristic to approximate the length of such *k*-mismatch substrings, and we describe *kmacs*, an efficient implementation of this idea based on generalized enhanced suffix arrays.

**Results:** To evaluate the performance of our approach, we applied it to phylogeny reconstruction using a large number of DNA and protein sequence sets. In most cases, phylogenetic trees calculated with *kmacs* were more accurate than trees produced with established alignment-free methods that are based on *exact* word matches. Especially on protein sequences, our method seems to be superior. On simulated protein families, *kmacs* even outperformed a classical approach to phylogeny reconstruction using multiple alignment and maximum likelihood.

**Availability and implementation:** *kmacs* is implemented in C++, and the source code is freely available at http://kmacs.gobics.de/

**Contact:** chris.leimeister@stud.uni-goettingen.de

**Supplementary information:** Supplementary data are available at *Bioinformatics* online.

## 1 INTRODUCTION

Comparative sequence analysis traditionally relies on pairwise or multiple sequence alignment. With the huge datasets that are produced by next-generation sequencing technologies, however, today’s alignment algorithms reach their limits. Thus, with the growing number of completely or partially sequenced genomes, there is an urgent demand for faster sequence-comparison methods. Over the past two decades, a wide variety of alignment-free approaches were proposed (Vinga and Almeida, 2003). Although aligning two sequences takes time proportional to the product of their lengths, most alignment-free methods run in linear time. They are, therefore, increasingly used for genome-based phylogeny reconstruction and for large-scale protein sequence comparison. It is known, however, that alignment-free methods are generally less accurate than alignment-based approaches.

Most alignment-free methods calculate the relative frequencies of words of a fixed length *k*, also called *k*-mers, in the input sequences. Other methods are based on variable-length matches; they have the advantage that it is not necessary to specify a fixed word length (Comin and Verzotto, 2012; Didier *et al.*, 2012). These programs achieve usually better results than approaches relying on a fixed word length. However, algorithms using variable word lengths are typically more complex and require more sophisticated data structures than methods relying on fixed word lengths.

A well-known approach that uses word matches of variable length is the *average common substring* (*ACS*) method (Ulitsky *et al.*, 2006), which calculates for each position *i* in one sequence the length of the longest substring starting at *i* and matching some substring of a second sequence. As a further development of this idea, the *shortest unique substring* (*shustring*) approach has been proposed by Haubold *et al.* (2005). These authors also derived an estimator for the number of substitutions per site between two unaligned sequences based on the average *shustring* length; they implemented this approach in the program *K_r_* (Haubold *et al.*, 2009). *ACS* and *shustrings* can be calculated efficiently using *suffix trees* (Weiner, 1973).

As the aforementioned methods, most approaches for alignment-free phylogeny reconstruction are based on *exact* word matches. Recently, we suggested to use *spaced-k-mers* defined by pre-defined patterns of *match* and *don’t care* symbols, instead of contiguous *k*-mers (Boden *et al.*, 2013; Leimeister *et al.*, 2014). The aim of this study is to apply the idea of *inexact* matches to word matches of varying lengths. We generalize the *ACS* approach by considering, for each position *i* in one sequence, the longest substring starting at *i* and matching some substring in the second sequence with *k* mismatches. We propose an efficient heuristic to approximate the lengths of these substrings, and we describe *kmacs*, an implementation of this approach based on *generalized enhanced suffix arrays*. A web server for our program is described in Horwege *et al.* (2014).

## 2 APPROACH

### 2.1 The *ACS* approach and *k*-mismatch substrings

As usual, for a sequence *S* over an alphabet Σ, *S*[*i*] is the *i*-th element of *S*, by |S| we denote the length of *S* and S[i..j] is the (contiguous) substring of *S* from *i* to *j*. In particular, S[i..|S|] is the *i*-th *suffix* of *S*. For two sequences *S*_1_ and *S*_2_, the *ACS* approach determines for every position *i* in *S*_1_ the length $s_{1}(i)$ of the longest substring of *S*_1_ starting at position *i* and exactly matching some substring in *S*_2_. The lengths $s_{1}(i)$ are averaged and normalized to define a similarity measure

(1) $$L(S_{1},S_{2})=\frac{1}{\left|S_{1}\right|}*\sum_{i=1}^{\left|S_{1}\right|}s_{1}(i)$$

which is turned into a (non-symmetric) distance measure by defining

(2) $$d(S_{1},S_{2})=\frac{log(|S_{2}|)}{L(S_{1},S_{2})}-\frac{log(|S_{1}|)}{L(S_{1},S_{1})}$$

To obtain a symmetric distance, the distance between *S*_1_ and *S*_2_ is then defined by Ulitsky *et al.* (2006) as

(3) $$d_{ACS}(S_{1},S_{2})=\frac{d(S_{1},S_{2})+d(S_{2},S_{1})}{2}$$

In this article, we generalize this distance measure by using substring matches with *k* mismatches instead of exact matches. That is, instead of using the maximum substring lengths $s_{1}(i)$, we define $s_{1}^{k}(i)$ as the length of the longest substring of *S*_1_ starting at position *i* and matching some substring of *S*_2_ with up to *k* mismatches, minus *k*. (We subtract *k* from the length of this string, counting only the *matching* positions). $s_{2}^{k}(i)$ is defined accordingly. We then define a distance measure as above, but with $s_{q}(i)$ replaced by $s_{q}^{k}(i)$. In the special case where *k* = 0, we have $s_{q}^{0}(i)=s_{q}(i)$, so in this case our distance is exactly the distance *d_ACS_*.

### 2.2 Approximating the length of k-mismatch substrings

For a pair of sequences, the exact values $s_{q}^{k}(i)$ can be calculated in $O(k*n^{2})$ time using suffix trees or similar data structures where *n* is the maximal length of the sequences. As we want to compare sequences in linear time, however, we propose a heuristic to approximate these values. To do so, we first calculate for each position *i* in *S*_1_ the length $s_{1}(i)$ of the longest common substring starting at *i* matching a substring of *S*_2_, as is done in *ACS*. Let *j* be the start of this matching substring in *S*_2_; the character $S_{1}[i+s(i)]$ must therefore differ from $S_{2}[j+s(i)]$. We then extend this match without gaps in *S*_1_ from position i+s(i)+1 and in *S*_2_ from j+s(i)+1, until the next mismatch occurs. This is repeated until the *k* + 1-th mismatch or the end of one of the two sequences is reached.

In the example below, for position *i* = 4 in *S*_1_ and with *k* = 2 mismatches, our approach would return the following *k*-mismatch common substring, starting at position *j* = 2 in *S*_2_:


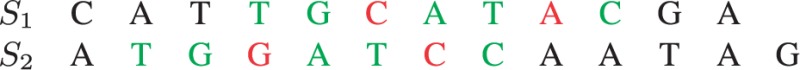


To obtain this *k*-mismatch common substring, our program would first determine the longest common substring for position *i* = 4 in *S*_1_ that exactly matches a substring in *S*_2_. We find such a match at position *j* = 2 in *S*_2_ with the length $s_{1}(4)=2$. Then this match is extended without gaps until the third mismatch is reached. The length of this 2-mismatch substring is 7, so we have $s_{1}^{2}(4)=5$ (in the definition of $s_{q}^{k}(i)$, we count only the *matching* positions).

It should be mentioned that, for a position *i* in *S*_1_, the corresponding position *j* in *S*_2_ of the longest exact match to a substring starting at *i* may not be unique. Consider, *e.g.* position *i* = 2 in the first sequence of the above example:


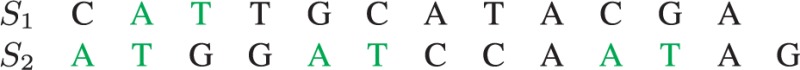


Here, the substring *AT* starting at position 2 in *S*_1_ is the longest substring starting at this position and matching a substring of *S*_2_—but this substring occurs at positions 1, 5 and 10 in *S*_2_. In such a case, we calculate *all k*-mismatch extensions of these occurrences as described above, and we define $s_{1}^{k}(i)$ as length of the *maximal* possible extension minus *k*.

The above heuristic reduces the complexity of finding the *k* mismatch maximal substring lengths from $O(k*n^{2})$ to O(k*n*z), where *z* is the average number of maximal matches to a substring in *S*_2_ starting at a position *i* in *S*_1_. In principle, this complexity could be achieved by using *suffix trees* (Weiner, 1973) as the underlying data structure. Here, one would build a *generalized suffix tree* for the sequences in $O(|S_{1}|+|S_{2}|)$ time, e.g. using *Ukkonen’s algorithm* (Ukkonen, 1995). To determine the longest substring starting at *i* in *S*_1_ and also occurring in *S*_2_, one needs to find the *lowest* node *v* in the suffix tree that is above leaf *i* and also above some leaf that belongs to *S*_2_. The length $s_{1}(i)$ of the longest common substring starting at *i* is then the *string depth* of the node *v*, that is, the length of the edge labels on the path from the root to *v*. Moreover, the leaves below *v* appertaining to *S*_2_ exactly correspond to the positions of this longest exact match in *S*_2_.

Next, we want to extend the longest exact matches that we have found by this procedure until the *k* + 1-th mismatch is found. Thus, we need be able to find the longest exact match between two sequences starting at two *given* positions *i* and *j* (the positions after a mismatch, in our case). In a suffix-tree approach, this could be accomplished by *lowest common ancestor* (*LCA*) queries. Similar to the aforementioned approach, we would have to look up the lowest node *v* that is above both leafs *i* and *j*; the string depth of *v* is then the length of the longest exact match starting at *i* and *j*, respectively. *LCA* queries can be carried out for any *i* and *j* in constant time after a linear-time preprocessing step (Harel and Tarjan, 1984), resulting in *k* constant-time *LCA* queries for the full *k*-mismatch extension of an exact longest match.

## 3 IMPLEMENTATION

Abouelhoda *et al.* (2004) have shown that every algorithm that uses suffix-trees can be replaced by an algorithm using *enhanced suffix arrays* that has the same complexity. Here, an *enhanced suffix array* is defined as a data structure ‘consisting of the suffix array and additional tables’. Both, suffix trees and enhanced suffix arrays, can be calculated in linear time and space, but suffix arrays require substantially less memory per input character than suffix trees do (Manber and Myers, 1990). In our implementation, we therefore used *enhanced* suffix arrays instead of suffix trees, making use of recent improvements of linear-time suffix array construction algorithms.

A *suffix array SA* of a string S=S[1]…S[n] is a permutation of the indices 1...n according to the lexicographical ordering of the corresponding suffices. That is, we have *SA*[i]=j if the *j*-th suffix of *S* is at the *i*-th position in the lexicographical ordering of all suffices of *S*. In addition to the *SA*, we need the so-called *longest common prefix (LCP) array* for *S*. Here, the entry *LCP*[*i*] stores the length of the *LCP* of the *SA*[*i*]-th suffix and its predecessor in *SA*, the *SA*[i−1]-th suffix. The *SA* of a sequence *S* together with the corresponding *LCP* array is called, in this context, the *enhanced suffix array* of *S*. To calculate *enhanced suffix arrays* in linear time, we used a program described by Fischer (2011), which is available at http://algo2.iti.kit.edu/english/1828.php. The underlying algorithm is based on *sais-lite* by *Yuta Mori*, a fast implementation of *induced sorting* (Nong *et al.*, 2009). Suffix arrays provide an efficient solution to our longest *k*-mismatch substring problem.

For a *single* sequence *S* and a position *SA*[*i*] in *S*, the enhanced suffix array of *S* can be used to find the length of the longest substring in *S* starting at a different position in *S* and matching a substring starting at *SA*[*i*]. It is easy to see that this substring must be the *LCP* of the *SA*[*i*]-th suffix with one of its neighbours in *SA*, i.e. either with the *SA*[i+1]-th or the *SA*[i−1]-th suffix, whichever is longer. With an *enhanced* suffix array, the length of this substring is given as the maximum of the values *LCP*[*i*] and LCP[i+1] and can therefore be looked up in constant time. The position where this second substring starts is then either *SA*[i−1] or *SA*[i+1]—or both of these positions—depending on where the maximum is reached.

**Fig. 1.**
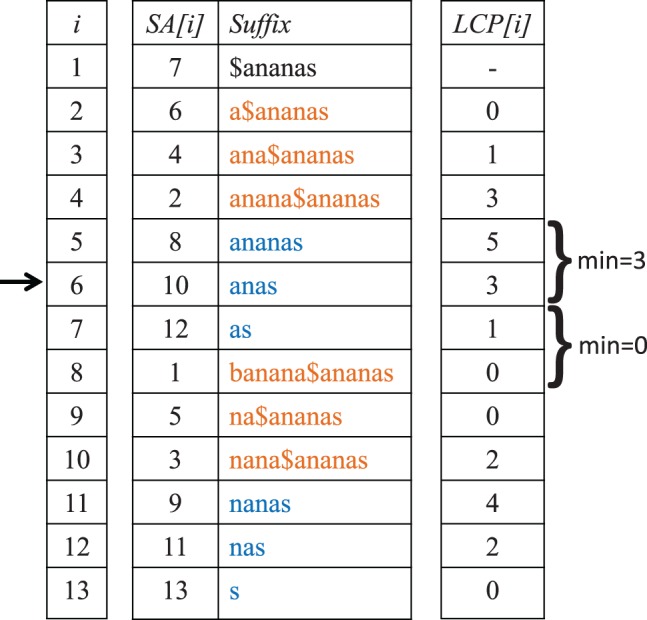
Generalized *SA* and *LCP* array for the strings $S_{1}=banana$ and $S_{2}=ananas$, concatenated by the symbol $. Suffices of $S_{1}\$S_{2}$ starting in *S*_1_ are shown in orange, suffices starting in *S*_2_ are in blue

If matches between two sequences are to be found, the situation is slightly more complicated. For a position in sequence *S*_1_, we want to find a position in *S*_2_ such that the common substring starting at these two positions is maximal, and vice versa. To solve this problem, we build the *generalized enhanced suffix array* of our sequences, i.e. the enhanced suffix array of the concatenated sequence $S:\;=S_{1}\$S_{2}$ where $ is a special character not contained the alphabet Σ; see also Babenko and Starikovskaya (2008) for a related approach. Thus, each suffix from *S*_1_ or *S*_2_ is represented in lexicographical order by an entry in *SA*. Figure 1 shows the enhanced suffix array for two sequences.

To find the length of the longest substring starting at *SA*[*i*] in one sequence, matching a substring of the *other* sequence, and its occurrences there, we need to look up the *largest* integer $p_{1}(i)$ with $p_{1}(i)<i$, such that *SA*$\left[p_{1}(i)\right]$ belongs to the other sequence. Correspondingly, we need the *smallest* integer $p_{2}(i)$ with $p_{2}(i)>i$ with *SA*$\left[p_{2}(i)\right]$ belonging to the other sequence. The length of this common substring is then given as the *minimum* of all *LCP* values between $p_{1}(i)+1$ and *i* or the minimum between the *LCP* values between *i* + 1 and $p_{2}(i)$—whichever minimum is larger. Formally, the length of the longest substring starting at a position *SA*[*i*] and matching a substring of the respective other sequence is given as follows:

(4) $$s(SA[i])=max(\underset{p_{1}(i)<x\leq i}{\min}LCP[x],\underset{i<y\leq p_{2}(i)}{\min}LCP[y])$$

with *p*_1_ and *p*_2_ defined as above.

The *position* of this longest substring in *S* is then *SA*$\left[p_{1}(i)\right]$ or *SA*$\left[p_{2}(i)\right]$ (or both), depending on where the maximum in Equation (4) is reached. All positions in this formula refer to the *concatenated* sequence *S*, but it is trivial to retrieve the positions in the original sequences *S*_1_ and *S*_2_ from these values by subtracting $|S_{1}|+1$ where necessary.

As an example, consider Figure 1. For *i* = 6, we want to find the longest common substring starting at SA[6]=10 (marked by an arrow) that exactly matches a substring starting at some position in the other sequence. Position SA[6]=10 in the concatenated sequence *S* corresponds to a position in sequence *S*_2_, so we have $p_{1}(6)=4$, as 4 is the largest integer smaller than 6 such that SA[4] belongs to the *other* sequence, i.e. to *S*_1_. Similarly, we obtain $p_{2}(6)=8$. According to Equation (4), we get the following:

s(SA[6])=max⁡{min⁡{5,3},min⁡{1,0}}=max⁡{3,0}=3.

Position 10 in *S* corresponds to position 3 in the original sequence *S*_2_, so, as a result, we obtain $s_{2}(3)=3$, i.e. the longest substring starting at position 3 in *S*_2_ matching a substring from *S*_1_ has length 3 (the substring itself is ‘*ana*’).

**Algorithm 1 Calculation of Equation (4)**

**Require:** SA {generalized suffix array for *S*_1_ and *S*_2_ of length *n*}

**Require:** LCP {corresponding longest common prefix array}

**Ensure:** *s* {stores the results of Equation (4)}

 min←0

 **for** *i* = 2 to *n* – 1 **do**

  **if** SA[i] and SA[i+1] belong to the same sequence **then**

   **if** LCP[i+1]<min **then**

    min←LCP[i+1]

   **end** if

   s[i+1]←min

  **else**

   min←LCP[i+1]

   s[i+1]←LCP[i+1]

  **end** if

 **end for**

 min←0

 **for** *i* = *n* to 2 **do**

  **if** SA[i] and SA[i+1] belong to the same sequence **then**

   **if** LCP[i]<min **then**

    min←LCP[i]

   **end** if

   s[i−1]←max(min,s[i−1])

  **else**

   min←LCP[i]

   s[i−1]←max(min,s[i−1])

  **end** if

 **end for**

All values *s*(*i*) can be calculated for the entire concatenated string *S* in *linear* time using Algorithm 1. Here, the first loop computes $\min_{p_{1}<x\leq i}LCP[x]$ for all indices *i* and stores them as *s*[*i*]. Then the second loop calculates $\min_{i<y\leq p_{2}}LCP[y]$ and updates *s*[*i*] if the result is greater than the actual value of *s*[*i*]. This way, algorithm 1 applies Equation (4) to all indices *i* and stores the corresponding values *s*[*i*].

Finally, for our heuristic we need to find for an index *i all* positions belonging to the respective other sequence, where a match of length *s*(*i*) occurs. This can be achieved by a simple extension of Algorithm 1. Without loss of generality, we assume that the first minimum in Equation (4) is strictly larger than the second minimum, so $p_{1}(i)$ is a position where a maximal match to the other sequence occurs (as was the case in our small example above). To find possible additional matching positions, we consider *all* indices $p\leq p_{1}(i)$ in descending order, as long as one has the following inequality:

$$LCP[p+1]\leq \underset{p_{1}(i)<x\leq i}{\min}LCP[x]$$

For *all* such *p* that belong to the other sequence, the positions *SA*[*p*] are occurrences of longest substrings matching a substring starting at *i*. In our example, we find one further position *p* = 3, so *SA*[3]=4 is an additional occurrence. If the maximum in (4) is achieved by the second term, one proceeds accordingly.

Next, the second step in our approach involves finding the length of the longest common substring starting at pre-defined positions in *S*_1_ and *S*_2_, respectively. Using the enhanced suffix array of a sequence *S*, the length of the longest substring starting at positions *SA*[*i*] and *SA*[*j*] (with *SA*[i]< *SA*[*j*]) is given as the minimum over the values *LCP*[*p*], i<p≤j. There is an approach similar to *LCA* queries to obtain this value known as *range minimum queries (RMQ)*. A *RMQ* returns the index of an array *A* that stores the smallest element between two specified indices *l* and *r*, denoted as $RMQ_{A}(l,r)$.

Several algorithms are available that can solve *RMQ* in constant time, after a linear preprocessing step, e.g. Fischer and Heun (2007). According to Fischer and Heun (2006), the longest common substring starting at *i* and *j* can be calculated as $LCP[RMQ_{LCP}(SA^{-1}[i]+1,SA^{-1}[j])]$ where $SA^{-1}$ is the inverse suffix array. As a result, the same complexity as for suffix trees can be achieved by using *enhanced suffix arrays*. In our implementation, however, we extend the substrings by matching single characters because in our test runs this ‘naive’ approach was faster than the *RMQ* implementation that we tested. Nevertheless, our downloadable program has an option for using the *RMQ* algorithm so the user can compare these two approaches.

## 4 BENCHMARKING

### 4.1 Benchmark sequences

To evaluate *kmacs* and to compare it with other methods of sequence comparison, we applied these methods phylogeny reconstruction. We used a large number of DNA and protein sequence sets for which reliable phylogenetic trees are available, and we measured how similar the constructed trees are to the respective reference trees. The following sequence sets were used in our study:

For eukaryotic DNA comparison, we used a set of 27 primate mitochondrial genomes that were previously used by Haubold *et al.* (2009) as benchmark for alignment-free methods. These sequences have a total length of 446 *kb*. A benchmark tree that has been constructed based on a multiple alignment.

As prokaryotic genomes, we used a set of 32 *Roseobacter* genomes, which were previously analysed by Newton *et al.* (2010). They constructed a phylogenetic tree for these sequences based on alignments of 70 universal single-copy genes that we used as reference tree in our study. The total size of this sequence set is 135.9 *mb*.

As benchmark proteins, we used 218 sequence sets contained in the *BAliBASE (v3.0)* database (Thompson *et al.*, 2005). To obtain reference trees, we applied *Maximum Likelihood* (Felsenstein, 1981), implemented in the program *proml* from *PHYLIP* to the *reference multiple alignments* in *BAliBASE*. As these reference alignments are considered to be reliable, the resulting trees should also be reliable.

In addition to these real-world sequences, we used the program *Rose* (Stoye *et al.*, 1998) to generate simulated DNA and protein families. *Rose* generates sets of related sequences based on a probabilistic model of substitutions and insertions/deletions for which the parameters can be adjusted by the user. These sequences are created along a randomly generated tree, starting from one common ancestral sequence at the root of the tree. This way, the ‘evolution’ of the generated sequences is logged, so a reference tree is generated alongside the sequences. We used *Rose* with default parameters, except for the parameter *relatedness*, which defines the average evolutionary distance between the generated sequences, measured in *PAM* units. We generated 20 DNA sequence sets, each of which contains 50 sequences with an average length of 16 000 *nt* using a *relatedness* value of 70. Furthermore, we generated 20 protein sequence sets, each containing 125 sequences with an average length of 300 *amino acids*. Here, we set the *relatedness* to 480.

### 4.2 Compared methods

We compared our new method with seven state-of-the-art alignment-free methods, namely *ACS* (Ulitsky *et al.*, 2006), *K_r_ v2.0.2* (Haubold *et al.*, 2009), *FFP* (Sims *et al.*, 2009), *spaced words* (Leimeister *et al.*, 2014), CVTree (Qi *et al.*, 2004), the *underlying approach (UA)* (Comin and Verzotto, 2012) as well as to a generic *k-mer*-frequency approach. As an eighth method, we ran *Clustal W* (Thompson *et al.*, 1994) on those sequence sets where this was possible and meaningful. For *ACS* and the *k-mer* approach, we used our own implementations, namely *kmacs* with *k* = 0 and our *spaced-words* approach without *don’t care* positions in the underlying patterns, respectively.

*FFP*, *K_r_* and *CVTree* return pairwise distances between the input sequences. For *ACS*, we calculated distances as defined in (3), and for *spaced words* and the *k-mer* approach we used the *Jensen–Shannon divergence* (Lin, 1991), applied to (spaced)-word frequency vectors as explained in Leimeister *et al.* (2014). For each of the five groups of benchmark data, we used the word length *k* for which the *k*-mer approach produced the best results, i.e. trees with minimal average *Robinson–Foulds* (*RF*) distances to the reference trees. For *spaced words*, we used the same value for *k*, even though better results might be possible with different values. Accordingly, on every group of benchmark data, we tested *FFP*, *CVTree* and *UA* with different parameter values and used those which produced the best results on this group.

We then constructed phylogenetic trees by applying *Neighbor joining* (Saitou and Nei, 1987) to the distance matrices obtained with the different alignment-free methods. Finally, we calculated phylogenetic trees for all sequence sets by applying *Maximum Likelihood* (Felsenstein, 1981) to the *Clustal W* multiple alignments. All resulting tree topologies were compared with the topologies of the respective reference trees using the *RF metric* (Robinson and Foulds, 1981). For *Neighbor joining* and to calculate the *RF* distances, we used the programs *neighbor* and *treedist* contained in the *PHYLIP package* (Felsenstein, 1989).

## 5 RESULTS AND DISCUSSION

**Fig. 2.**
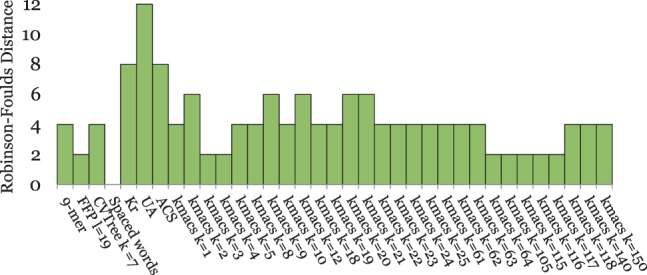
Performance of alignment-free methods on a set of 27 primate mitochondrial genomes. *RF* distances between constructed trees and a reference tree are shown. The tree calculated by *kmacs* with *k* = 70 is shown in Figure 3, together with the reference tree

Figures 2 and 4–7 summarize our test results on the five groups of benchmark sequence sets that we used. The plots show the average *RF* distances between the produced trees and the corresponding reference trees. For *kmacs*, results are shown for various values of *k*. For *FFP*, *CVTree*, *UA* and the *k*-mer method, we also used a range of parameter values, but for each of these methods, the figures show only the *best* results on the respective group of benchmark sequences. Thus, for a fair comparison, these methods should be compared with the *best* results of *kmacs* in the corresponding figure. On the other hand, *K_r_*, *ACS* and *Clustal* could be used with default parameters, which is clearly an advantage of these methods.

**Fig. 3.**
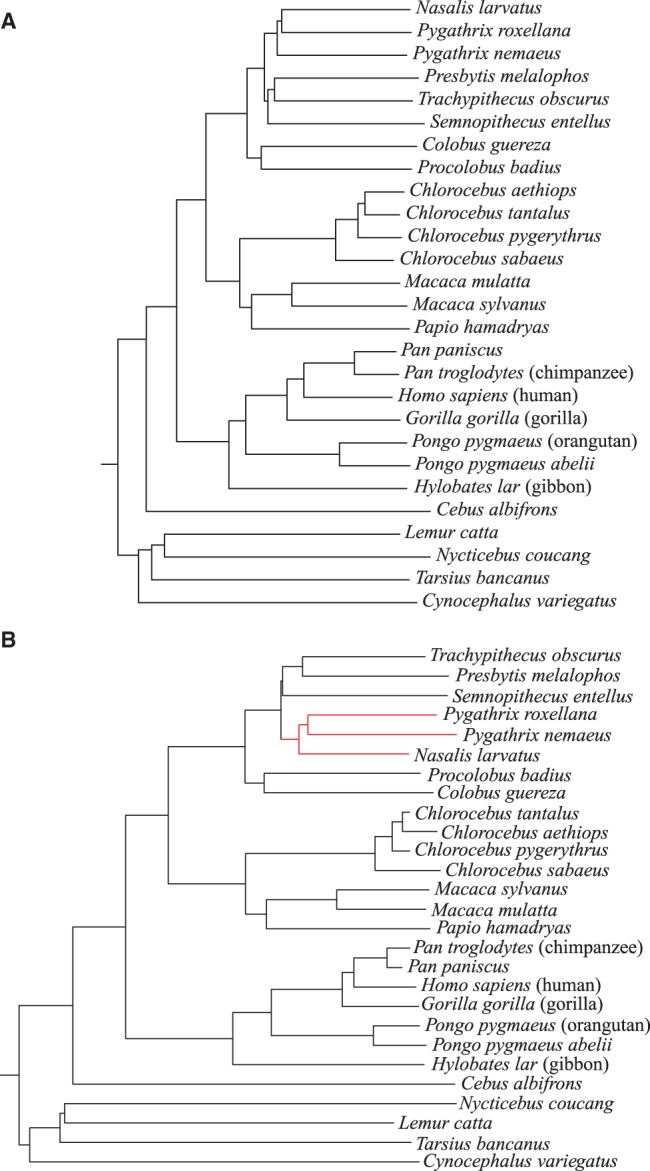
Midpoint-rooted trees of 27 primate mitochondrial genomes. (**A**) is the alignment-based reference tree obtained from Haubold *et al.* (2009) and (**B**) is based on *kmacs* with *k* = 70. Red branches represent differences to the reference tree topology. Except for these three species, the topologies of the two trees coincide, resulting in a *RF* distance of 2 between our tree and the reference tree

Figure 2 contains the test results on the primate mitochondrial genomes. The best method on this dataset was our previously developed *spaced-words* approach; the tree topology produced by this method precisely coincides with the topology of the reference tree, i.e. the *RF* distance is zero. The second best methods were *FFP* and *kmacs* with *k* = 3, 4 and 64≤k≤117. *ACS*, *CVTree*, *UA*, *kmacs* with other values for *k* and *K_r_* performed worse on these data. As an example, Figure 3 compares the tree calculated with *kmacs* (*k* = 70) with the alignment-based reference tree from Haubold *et al.* (2009). The tree topology calculated by *kmacs* almost coincides with the topology of the reference tree; the *RF* distance between these trees is 2.

**Fig. 4.**
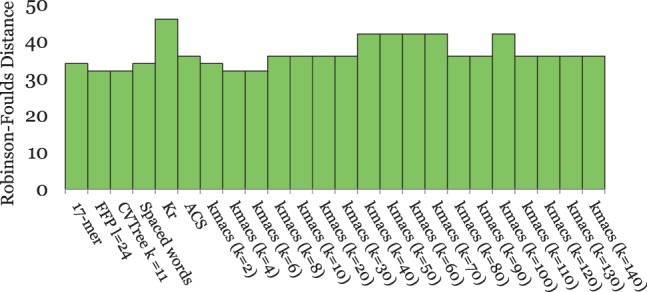
Performance of alignment-free methods on a set of 32 *Roseobacter* genome sequences. *RF* distances to the reference tree are shown

**Fig. 5.**
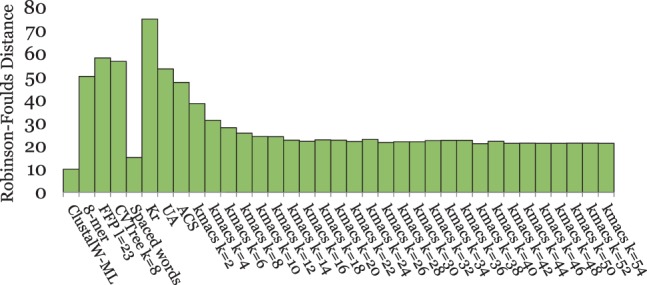
Performance of alignment-based and alignment-free methods on 20 sets of 50 simulated DNA sequences of length 16 000 each. Average *RF* distances to the respective reference trees are shown

On the *Roseobacter* genomes, the best methods were *kmacs* with *k* = 4 and 6, *FFP* and *CVTree* as shown in Figure 4. *Spaced words* and the generic *k*-mer approach performed slightly worse. None of the tested methods was able to exactly reconstruct the topology of the reference tree. *UA* is missing in this comparison, as this program is too slow to be run on full bacterial genomes in reasonable time. For our simulated DNA sequence sets, the results were similar as for the primate mitochondrial genomes; see Figure 5. Here too, *spaced words* was the best alignment-free method, followed by *kmacs*. This time *kmacs* outperformed the established alignment-free approaches for *all* values of *k* that we tested. On our simulated DNA sequences, we could also run a classical approach to phylogeny reconstruction using *Clustal W* and *Maximum Likelihood*. Not surprisingly, this slow and accurate method performed better than all alignment-free approaches.

**Fig. 6.**
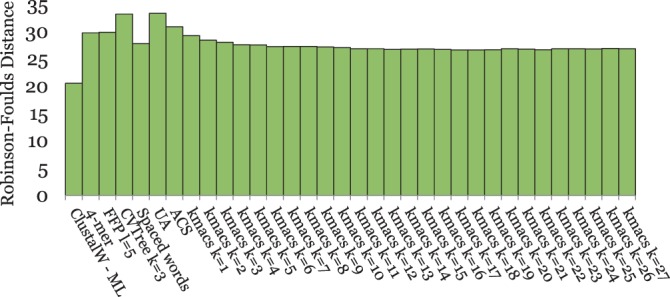
Performance of different methods on 218 protein sequence sets from *BAliBASE*. Average *RF* distances to reference trees, calculated based on *BAliBASE* reference alignments, are shown

Figure 6 shows the results for the *BAliBASE* protein sequences. *Spaced words* and *kmacs* again produced better results than the existing alignment-free methods that we evaluated. This time, there was a large range of values for *k* where *kmacs* performed similar or even slightly better than *spaced words,* and both methods outperformed the other alignment-free methods that we tested. As with the previous dataset, the classical approach based on multiple sequence alignment performed best; this time the difference between alignment-based and alignment-free methods was larger. This may be because of the fact that multiple-alignment programs are often tuned to perform well on *BAliBASE*, the main database to evaluate multiple-alignment methods.

**Fig. 7.**
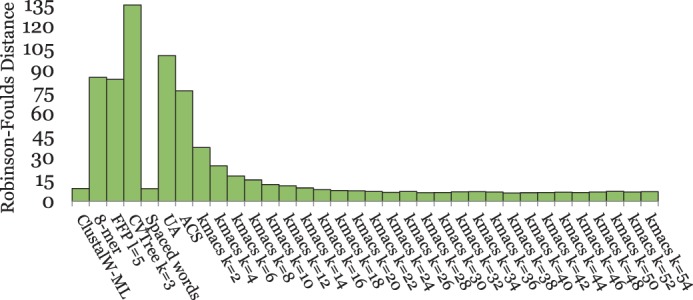
Performance of different methods on 20 sets of 125 simulated protein sequences each

Finally, the results on our simulated protein sequences are shown in Figure 7. As in most previous examples, *spaced words* and *kmacs* outperformed other alignment-free approaches and, as on *BAliBASE*, *kmacs* was slightly better than *spaced words* if *k* was sufficiently large. Surprisingly, on these benchmark sequences *spaced words* and *kmacs* even outperformed *Clustal W* and *Maximum Likelihood*, although not dramatically.

**Fig. 8.**
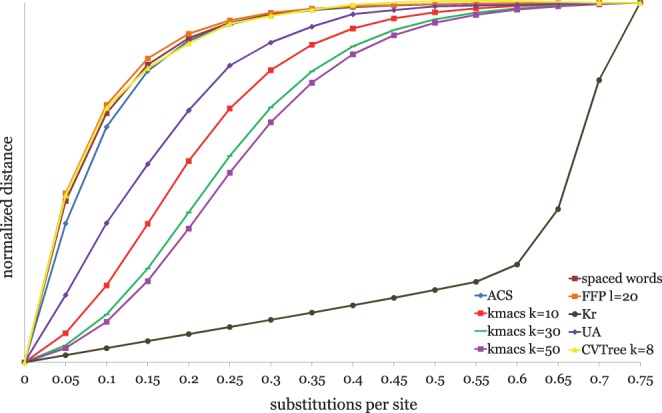
Distances calculated by different alignment-free methods as a function of substitutions per site for pairs of simulated DNA sequences. Distances were normalized such that they are equal for 0.75 substitutions per site

So far, we evaluated alignment-free and alignment-based methods *indirectly*, by applying them to phylogeny reconstruction and comparing the resulting trees with trusted reference trees using the *RF* metric. This is a common procedure to evaluate alignment-free methods. *RF* distances to reference trees are only a rough measure of accuracy, though, as they are based on tree topologies alone and do not take branch lengths into account. Furthermore, the constructed trees depend not only on the underlying methods for sequence comparison but also on the methods used for tree reconstruction. A more direct and accurate way of comparing alignment-free methods is to *directly* compare the distance values that they calculate. This can be done, for example, by plotting the distances produced for simulated sequences against their *real* evolutionary distances (Haubold *et al.*, 2009). Ideally, this should be a linear relation. Figure 8 shows such plots for the algorithms that we compared in our study.

Tables 1 and 2 summarize the run times of the different methods that we tested. When used with moderate values of *k*, *kmacs* is faster than *spaced words* run with a set of 100 different patterns. *K_r_* was more than one order of magnitude faster than *kmacs* and *spaced words*, respectively, although *UA* was the slowest method. The fastest method was our implementation of the generic word-frequency approach, followed by *K_r_* and *CVTree*. In general, *spaced words* used with the *single-pattern* option is only slightly slower than the *k*-mer approach. As shown in our companion paper, however, *spaced words* produces considerably better results when used with *multiple* patterns (Leimeister *et al.*, 2014). We therefore applied only the multiple-pattern version in this study.

**Table 1.** Program runtime for different methods on a set of 50 simulated DNA sequences of length 16 000 *nt* each

Method	Runtime (*s*)
*Clustal W*	1817
*Clustal* Ω	1039
8-mer	0.3
*FFP*, *l* = 23	123.3
*spaced words*, 100 patterns, *k* = 8	27.6
*ACS*	2.8
*K_r_*	0.9
CVTree	0.5
UA	572
*kmacs*, *k* = 1	4.2
*kmacs*, *k* = 10	7.6
*kmacs*, *k* = 20	4.2
*kmacs*, *k* = 50	21.4

*Note: Spaced words* was run with 100 random patterns of varying length as described by Leimeister *et al.* (2014). For *Clustal W* and *Clustal* Ω, the time for calculating a multiple alignment is shown; for the six alignment-free methods the time for calculating pairwise distances is shown.

**Table 2.** Program run time for different methods on a set of 32 genome sequences of total length 135 *mb* from various *Roseobacter* species

Method	Runtime (*s*)
17-mer	34.9
*FFP*, *l* = 24	9022
*Spaced words*, 100 patterns, *k* = 17	3617
*ACS*	531
*K_r_*	206
CVTree	84
*kmacs*, *k* = 1	784
*kmacs*, *k* = 10	1302
*kmacs*, *k* = 50	3158
*kmacs*, *k* = 100	5433

*Note:* Parameters for *spaced words* as in Table 1.

The relatively long runtime of *UA* is partially because of the fact that this program is written in *Java*, while all other programs that we tested are written in *C++*. As expected, the multiple-alignment approaches *Clustal W* and *Clustal* Ω (Sievers *et al.*, 2011) were far slower than the alignment-free methods; the difference in speed between alignment-based and alignment-free methods was between three and four orders of magnitude. All test runs were done on a *Intel Core i7 4820k*, which we overclocked to *4.5Ghz*.

**Fig. 9.**
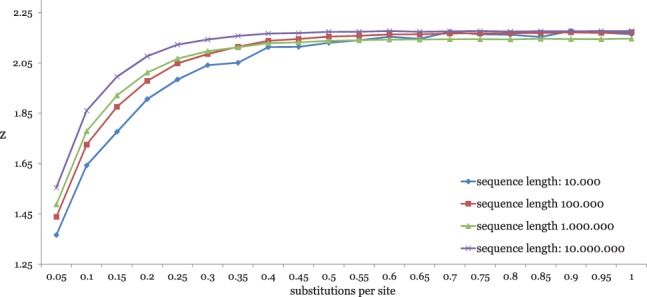
Average number *z* of maximal exact matches starting at a position *i* in one sequence to a substring in a second sequence. We used simulated DNA sequences with different lengths and substitution frequencies

As explained in Section 2.2, *kmacs* searches for each position *i* in one sequence the *maximum* substring starting at *i* that matches a substring in the second sequence. There can be more than one such maximal match, and *all* these matches are extended to *k*-mismatch common substrings. Thus, the runtime of *kmacs* depends on *z*, the average number of such maximal substring matches for a given position *i*. In principle, *z* can be large and the *worst-case* time complexity of our algorithm is therefore high. In practice, however, *z* is small, independent of sequence length and substitution probability. Figure 9 shows values of *z* for different sequence lengths and mutation frequencies.

**Fig. 10.**
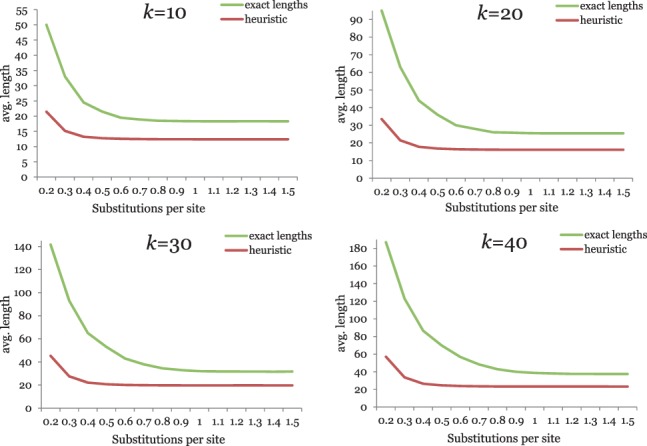
Average common *k*-mismatch substring lengths depending on the substitution frequency in simulated DNA sequences, estimated with our greedy heuristic (lower curve) and calculated with an exact algorithm (upper curve) for various values of *k*

Finally, we wanted to know how accurately our greedy heuristic approximates the *exact* maximal *k*-mismatch substring length. Figure 10 compares the average maximal *k*-mismatch substring length for varying substitution probabilities (*a*) as estimated with our heuristic and (*b*) calculated with a slow and exact algorithm. The figure shows that our heuristic is clearly suboptimal. But the goal of our project was not so much to precisely estimate the maximal *k*-mismatch substring lengths, but rather to define a distance measure on sequences that can be efficiently calculated and that can be used to obtain biologically meaningful results. Therefore, we think that the discrepancies between the optimal substring lengths and the values estimated by our heuristic are acceptable. Figure 10 suggests, however, that better estimates of the *k*-mismatch common substring lengths might improve the sensitivity of *kmacs* on divergent sequence sets because the curves for the exact solutions converge at higher substitution frequencies. In fact, on the mitochondrial genomes that we used as benchmark data, an exact algorithm led to better phylogenetic trees than our greedy heuristic (Supplementary Material). Therefore, it may be worthwhile to develop heuristics that approximate the maximal *k*-mismatch substring lengths more accurately.

## 6 CONCLUSION

Most alignment-free approaches to sequence analysis are based on *exact* word matches. In this article, we presented a novel alignment-free algorithm that takes mismatches into account. This is similar in spirit to the *spaced-words* approach that we previously proposed (Leimeister *et al.*, 2014). But while *spaced words* uses word pairs of a *fixed* length with possible mismatches at pre-defined positions, *kmacs* considers *maximal* substring matches with *k* mismatches at arbitrary positions. In the *spaced-words* approach, the number of *match positions* in the underlying patterns is a critical parameter for the performance of the method. In contrast, in *kmacs*, there seems to be a fairly large range of values for *k* that lead to high-quality results, as shown by our test results. *kmacs* seems therefore less sensitive to user-defined parameters.

The implementation of our approach using *generalized enhanced suffix arrays* enables us to analyse large sequence sets efficiently. Still, the program *K_r_* is roughly one order of magnitude faster than *kmacs*. One reason for this is that *K_r_* uses *one single* generalized suffix tree representing *all* input sequences, which can be calculated in time proportional to the number of sequences (Domazet-Lošo and Haubold, 2009). In contrast, *kmacs* calculates one generalized enhanced suffix array for *each pair* of sequences, so its run time is *quadratic* in the number of sequences. On the other hand, calculating suffix arrays for two sequences at a time is less memory consuming, as one does not need to keep the suffix array for *all* input sequences simultaneously in main memory. Thus, our approach can be applied to larger datasets than *K_r_*.

The two approaches that we developed, *kmacs* and *spaced words*, are slower than the corresponding approaches based on exact matches, *ACS* and the generic *k*-mer approach. Our new approaches, however, produce significantly better results than those established methods. Our test results suggest that *spaced words* performs slightly better than *kmacs* on genomic sequences, whereas on protein sequences, *kmacs* is superior.

In our program evaluation, we used DNA sequence sets with large evolutionary distances. On these sequences, our new alignment-free methods performed better than established methods that rely on exact word matches. Algorithms using exact matches, on the other hand, seem to work better on smaller evolutionary distances. *K_r_*, for example, performs best on evolutionary distances of up to 0.6 substitutions per site (Haubold *et al.*, 2009). Similarly, we observed that on closely related DNA sequences, *kmacs* produces sometimes best results with *k* = 0, i.e. without mismatches (unpublished results). It seems therefore best to apply *kmacs* to distantly related sequence sets, while methods such as *K_r_* and *ACS* may be preferred on evolutionarily more closely related sequences.

In biological sequences, substitutions are more frequent than insertions and deletions. Consequently, *exact* matches between local homologies can usually be extended until the first *substitution* is reached. The average length of longest common substrings and of shortest unique substrings, respectively, can therefore be used to estimate *substitution probabilities* (Haubold *et al.*, 2009). This is similar for *kmacs* as long as *k* is small enough. In this case, all *k* mismatches are likely to be used up in a *k*-mismatch common substring extension *before* the first indel occurs. Thus, the average length of the longest *k*-mismatch common substrings depends on the frequency of mismatches and could be used to estimate substitution probabilities, just as in *K_r_*.

In contrast, if *k* is sufficiently large, substring matches between local homologies are essentially extended until the first *indel* occurs. From this point on, the mismatch frequency is high and the remaining mismatches will be used up quickly. So in this situation, the average *k*-mismatch substring length depends on the frequency of *indels* rather than on the frequency of substitutions. This may explain why *ACS* and *K_r_* work well on closely related sequences, while *kmacs* is superior on distantly related sequences where the frequency of indels may be a better measure for evolutionary distances than the frequency of mismatches.

In our study, we used alignment-free methods to reconstruct phylogenetic trees and evaluated the quality of these trees. But phylogeny reconstruction is only one important application of sequence comparison. Clustering, classification and remote-homology detection are other fundamental challenges in DNA and protein sequence analysis. With the rapidly growing size of sequence databases, alignment-free methods have become indispensable for these tasks (Comin and Verzotto, 2012; Hauser *et al.*, 2013; Lingner and Meinicke, 2006). Given the speed of *kmacs* and the quality of the phylogenetic trees that we could produce with it, our approach should be useful not only for fast phylogeny reconstruction, but also for other tasks in comparative sequence analysis.

## Supplementary Material

Supplementary Data
