# Supplementary material for: kmacs: the k-mismatch average common substring approach to alignment-free sequence comparison
Source: Bioinformatics. 2014 May 13;30(14):2000–8. doi: 10.1093/bioinformatics/btu331 (PMC4080746; doi:10.1093/bioinformatics/btu331)
Supplement: Supplementary Data [file supp_30_14_2000__index.html]

kmacs: the k-mismatch average common substring approach to alignment-free sequence comparison — kmacs: the k-mismatch average common substring approach to alignment-free sequence comparison — Supplementary Data 

# kmacs: the *k*-mismatch average common substring approach to alignment-free sequence comparison
